# Molecular Characterization of Nonvolatile Fractions of Algerian Petroleum with High-Resolution Mass Spectrometry

**DOI:** 10.1021/acs.energyfuels.1c00333

**Published:** 2021-05-06

**Authors:** Fatima Saad, Boumedienne Bounaceur, Mortada Daaou, Juan Ramón Avilés-Moreno, Bruno Martínez-Haya

**Affiliations:** † LCPM, Département de Chimie, Faculté des Sciences Université d’Oran 1 (Ahmed Benbella), P.O. Box 1524 el m’naouer, Oran 31000, Algeria; ‡ LSPBE, Département de Génie Chimique, Faculté de Chimie, Université des Sciences et de la Technologie d’Oran- Mohamed Boudiaf, P.O. Box 1505 el m’naouer, Oran 31000, Algeria; § Department of Applied Physical Chemistry, Universidad Autónoma de Madrid, 28049 Madrid, Spain; ⊥ Department of Physical, Chemical and Natural Systems, Universidad Pablo de Olavide, 41013 Seville, Spain

## Abstract

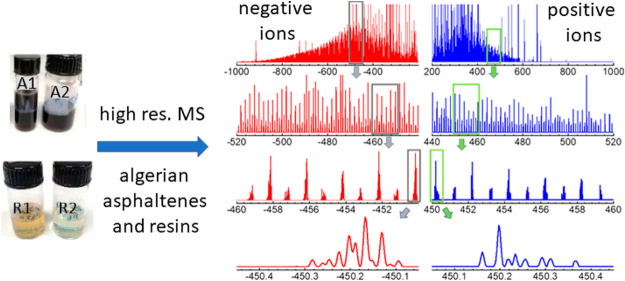

Algerian crude oil displays a marked propensity for asphaltene precipitation, leading to solid deposits during extraction, transportation, and storage. The relationship between precipitation and chemical composition is unclear; in fact, Algerian crude oil actually features a low asphaltene concentration, despite its relatively large rate of deposit formation. The rationalization of the precipitation process and its remediation should benefit from a molecular characterization of the crude oil. In this study, two unstable asphaltene fractions (A1 and A2) from two different deposits, and two resin crude oil fractions (R1 and R2) from the Hassi-Messaoud Algerian field have been characterized at the molecular level by means of high-resolution mass spectrometry with an Atmospheric Pressure Chemical Ionization (APCI) source. Positively and negatively charged compounds with molecular weights 200–1200 *m*/*z* were detected. Several thousand molecular stoichiometries were identified and classified for each sample, in terms of heteroatom content and aromaticity, searching for trends characteristic of the two asphaltenes and of the associated resins. The A2 asphaltene, from a downstream storage tank, displays a higher aromaticity and O-heteroatom content, which correlates with an enhanced aggregation propensity, in comparison to the A1 fraction, collected at the well bore. The resin fractions are found to be abundant in aliphatic hydrocarbons and heteroatomic compounds of moderate aromaticity. The more polar resin fraction, R2, is enriched in N-containing species, with respect to the less polar resin fraction R1, which correlates with the stabilizing function observed in previous works. The results stress the view of crude oil fractions as complex mixtures, rather than in terms of average prototypical compounds, when facing the understanding of asphaltene deposition conditions.

## Introduction

Asphaltenes are an extremely complex mixture of polyaromatic compounds, encompassing the heaviest and most polar fraction of crude oil.^1^ The molecular complexity of asphaltenes is a direct consequence of their phenomenological definition, in terms of solubility properties in aromatic solvents (toluene) versus light paraffinic solvents (e.g., *n*-heptane or *n*-hexane). There is solid experimental evidence supporting the theory that asphaltenes plausibly extend over molecular weights within the range of 200–2000 g/mol, and that they consist of polyaromatic species with varying abundances of condensed island-like and cross-linked archipelago-like structures and of heteroatom content (primarily oxygen, nitrogen, and sulfur).^2−6^ In addition, trace amounts of metals such as Ni, Fe, and V are present in asphaltenes, potentially contributing to their physicochemical properties.^7^

The extensive efforts devoted in the past decades to the elucidation of the molecular nature of asphaltenes and their related colloidal behavior relies on the enormous problems that they cause during the production, transportation, storage, and refining of crude oil. Asphaltenes are prone to precipitation and formation of solid deposits, eventually leading to plugging of pipelines and even wellbores, with significant costs related to remediation and loss of production. Consequently, oil companies invest significant budgets in chemicals that prevent asphaltene deposition.^8^ For instance, in the Hassi–Messaoud fields, more than 80 wt % of the asphaltenes in the crude oil eventually become incorporated into deposits in the tubing, which demand frequent washing treatments and injection of solvents and dispersants.^9,10^

The mechanisms leading to asphaltene deposit formation are complex and are not fully understood to date. Pressure, temperature, and compositional changes in the oil may have nontrivial effects on asphaltene precipitation.^11^ The molecular structure and the interactions of asphaltenes with other constituents of crude oil are considered important sources of modulation of their stability or precipitation. It has been suggested that asphaltenes with a higher polarity (higher heteroatom content) have a tendency to be more unstable, with respect to aggregation.^12−20^ Resin molecules are considered stabilizing agents that bind efficiently to asphaltenes, presumably preventing the growth of incipient aggregates.^21−34^ The presence of polar heteroatom groups and of small aromatic moieties in resins appears to pose the largest deterring effects on asphaltene precipitation.^35−38^

Therefore, the molecular characterization of asphaltenes and resins seems to be key to rationalizing aggregation and flocculation processes in crude oils. High-resolution mass spectrometry stands out among other analytical techniques, because of its ability to define the elemental composition and structure of crude oil constituents.^1^ Molecular stoichiometries (e.g., C*_c_*H_*h*_N_*n*_O_*o*_S_*s*_) are determined from the accurate measurement of exact masses. Structural information may be inferred from mass sequences and trends associated with heteroatom content, assisted by fragmentative MS/MS techniques.^2−5^ The consolidation of commercial Fourier transform–ion cyclotron resonance (FT-ICR), orbitrap and time-of-flight (TOF) mass analyzers, in combination with a range of ionization techniques, such as electrospray ionization (ESI), atmospheric pressure photoionization (APPI), atmospheric pressure chemical ionization (APCI), or laser desorption/ionization (LDI), has largely contributed to the recent advances in petroleum science.^1−5,39−51^ Among them, orbitrap mass spectrometry is currently being considered as a cost-effective benchtop high-resolution technique and it is finding increasing application in the field of petroleomics.^39,43−46^

In this paper, APCI-orbitrap mass spectrometry has been employed to characterize resin fractions and asphaltene deposits of Algerian crude oil from the Hassi-Messaoud fields. This crude oil constitutes a paradigmatic case of a large precipitation tendency, in apparent contrast with a comparably poor asphaltene content, of <1 wt %.^9^ Several studies have described the physicochemical behavior of Algerian crude oils and have evaluated their response to flocculants and emulsion stabilizers.^34,52−56^ Despite such rich literature around the precipitation problem, few previous studies have addressed the detailed characterization of nonvolatile fractions (i.e., asphaltenes and resins) of Algerian crude oils at a molecular level.^52,57,58^ A first incursion of our groups into the composition of Algerian asphaltenes, based on LDI mass spectrometry in combination with spectroscopic methods (FT-IR and NMR), showed that the structural properties of asphaltenes present in petroleum may vary significantly during the different stages of extraction, treatment, transportation and storage.^19,59^ A detailed correlation of crude oil deposits formed during those processes with compositional and structural changes (aromaticity, polarity and heteroatom content, etc.) was pending, because of the limited mass resolution of the TOF mass spectrometer employed.

This study focuses on the analysis of two differentiated crude oil deposits: one that is formed at the wellbore, plausibly associated with the gas-enhanced oil recovery employed in the field, and a second one that is formed downstream after extensive treatment and transportation of the oil. Profound differences are found between the composition of the asphaltenes extracted from the two deposits, which correlate with their aggregation propensity. The resins were characterized based on their potential role as stabilizers against asphaltene precipitation.

Consistent with previous high-resolution mass spectrometry investigations, the present results stress the importance of understanding crude oil fractions as complex mixtures, polydispersed in composition and structure, rather than viewing them in terms of average prototypical compounds.

## 2 Materials and Methods

Crude oil samples and two asphaltene deposits from the Algerian Hassi-Messaoud petroleum field were supplied by the Sonatrach Company. General characteristics of Hassi-Messaoud petroleum are summarized in Table 1.

**Table 1:** General Physical Characteristics of the Crude Oil from the Hassi–Messaoud Well^a^

parameter	value
gravity	45° API
viscosity at 40 °C	2.23 cP
total acidity	0.96 mg KOH/g
resin content	24.5% (w/w)
asphaltene content	0.70 (w/w)

a Data taken from ref (20).

The first deposit (DP1) was formed at the wellbore during enhanced oil recovery based on the injection of a gas mixture of C1–C5 hydrocarbons with 10% CO_2_. The second deposit of the same oil (DP2) was formed at the entrance of a downstream storage tank, to which the crude oil was transported after degassing and treatment for the removal of salts and water.

Toluene (99% purity), CS_2_ (99% purity), acetone, methylene chloride (99% purity), and dithranol (MALDI matrix, 99% purity) were supplied by Sigma–Aldrich, while *n*-hexane and *n*-heptane (98% purity) were purchased from Biochem Chemicals.

### 2.1 Extraction of Asphaltene and Resin Fractions

**Figure 1:**
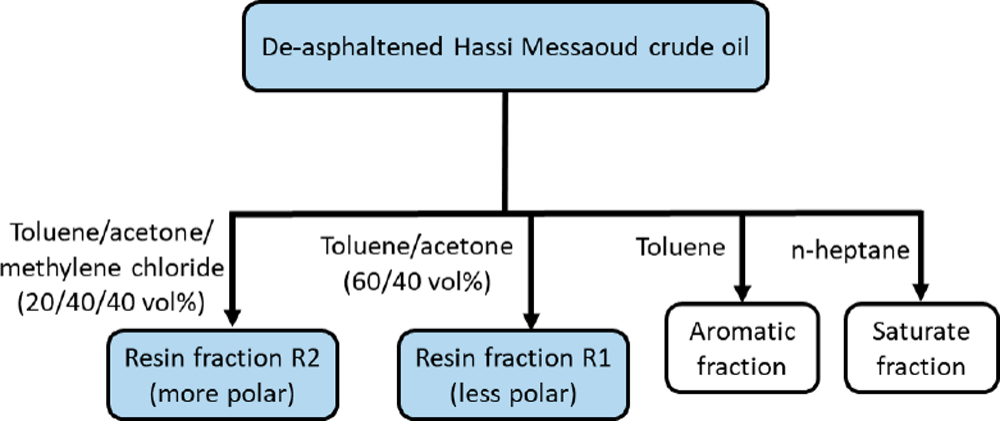
Schematic layout of the protocol employed for the extraction of resin fractions.

The extraction of the resin fractions was performed on crude oil collected at the wellbore, according to the ASTM D-2007 guidelines^60^ outlined in Figure 1. As described in detail elsewhere,^34^ the protocol fractionates deasphaltened oil into saturates, aromatics, and two resin fractions, namely, a low polarity resin fraction and a high polarity resin fraction, which will be referred to as R1 and R2, respectively.

Asphaltenes from deposit DP1 were extracted with either *n*-heptane (C_7_-A1) or *n*-hexane (A1) as flocculant. Asphaltenes from deposit DP2 (A2) were extracted with *n*-hexane. In all cases, the flocculant was added in an excess of 40:1 (cm^3^/g) ratio to the crude oil deposit. The mixture was stirred for 24 h in darkness at ambient temperature, and subsequently allowed to precipitate for additional 24 h. Finally, the precipitated asphaltenes were retained in filter paper with a pore diameter of 45 μm, and then dissolved in toluene. After evaporation of the solvent, asphaltenes were washed several times with fresh flocculant, until a visually clean supernatant was obtained. The extraction procedure resulted in recovery yields of 88 wt % and 85 wt % for the *n*-hexane asphaltenes A1 and A2, respectively, and of 60 wt % for the *n*-heptane asphaltene C_7_-A1.

### 2.2 Elemental Analysis of Asphaltene and Resin Fractions

Elemental composition (carbon, hydrogen, nitrogen, and sulfur) of the asphaltene and resin fractions were determined with a CHNS TruSpec Micro (LECO) elemental analyzer. Sulfur content was confirmed by inductively coupled plasma (optical ICP) quantification. The results of the elemental analysis of the A1, C_7_-A1, A2, R1, and R2 fractions are listed in Table 2, and they are discussed in Section 3.1. Given the apparently anomalous O content of asphaltene A2, we analyzed all samples in an independent facility in Seville, which delivered statistically coincident results.

**Table 2:** C, H, N, and S Composition (Weight Percentage), and Associated H/C Atomic Ratio of the Asphaltene and Resins Extracts Included in This Investigation

	Composition (Weight Percentage)					
sample	C	H	N	S	O^a^	H/C
A1	87.7	7.7	0.86	0.42	3.3	1.05
C_7_-A1	88.5	7.9	0.86	0.40	2.5	1.07
A2	79.5	6.0	0.14	0.41	13.9	0.90
R1	84.3	12.6	0.10	0.85	2.1	1.79
R2	77.6	10.9	0.35	1.40	9.7	1.68

a Oxygen evaluated from the mass balance.

### 2.3 Aggregation Onsets of the Asphaltene Fractions

To compare the stability of the asphaltene fractions, the aggregation onset point (AGO) and the precipitate content (wt %) of each asphaltene fraction were determined, using conventional methods based on ultraviolet–visible (UV-vis) spectrophotometry and gravimetry.^19^ In particular, absorbance at 750 nm was monitored as flocculant was added to toluene solutions of the asphaltenes, searching for light scattering effects associated with precipitated asphaltene particles.

### 2.4 APCI-Orbitrap Mass Spectrometry

The molecular analysis of asphaltenes and resins was conducted using a Q-Exactive Focus Hybrid Quadrupole-Orbitrap Mass Spectrometer (Thermo-Fisher) that was equipped with an APCI ion source. APCI is based on the nebulization of the sample and the generation of a corona discharge, yielding a chain of charge/proton transfer reactions, eventually ionizing the analytes.^61^ It is an efficient ionization technique for thermally stable molecular species of moderate size (up to 2000 amu) with medium to high polarity.^62^ In the present experiments, positive and negative ion mass spectra were acquired in full scan mode over the absolute mass range 200–1200 *m*/*z*, at a nominal resolving power of *M*/Δ*M* = 60 000 at *m*/*z* 300. Hence, the full width at half-maximum (fwhm) values of the individual peaks in the mass spectra ranged from 0.002 at *m*/*z* 300 to 0.007 at *m*/*z* 800, and the mass accuracy was of 5 ppm, as tested with different polyaromatic hydrocarbon calibration standards. The samples to be analyzed (asphaltenes or resins) were dissolved in carbon disulfide (CS_2_) at 1 mg mL^–1^ concentration and then sonicated for 20 min. Using CS_2_ as a solvent provided stronger signals than other organic solvents, e.g., toluene, because of a more efficient sample ionization in the APCI source, which is consistent with previous works.^63−65^ The solutions were directly infused into the APCI source at a flow rate of 50 μL min^–1^. The discharge current was set at 5 μA in all measurements. The temperature of the inlet capillary in the ionization source was maintained at 275 °C, while the temperature of the APCI vaporizer was adjusted for maximum ion signal to 300 and 450 °C in the positive and negative ion modes, respectively. Increasing the temperature for the positive-ion mode measurements deteriorated the signal without an appreciable trend of detection of a broader range of chemical species. Test measurements with lower asphaltene concentrations, down to 0.2 mg mL^–1^, did not yield evidence for changes in the ion distribution, because of potential signal suppression induced by aggregation effects.^2^

### 2.5 LDI-TOF Mass Spectrometry

Laser desorption ionization mass spectra with time-of-flight mass discrimination (LDI-TOF) of the asphaltenes and resins were collected in a Bruker-Daltonics UltrafleXtreme mass spectrometer, equipped with a 355 nm Nd:YAG laser. The laser pulse energy was set at 20% above the detection threshold of each sample (∼5 μJ), and 2500 laser shots were accumulated to produce each spectrum at a laser firing rate of 500 shots s^–1^. Positive-ion spectra were recorded in linear time-of-flight mode, leading to a mass resolution of *M*/Δ*M* = 6000 at *m*/*z* 300. The instrument was calibrated using external polyaromatic and polydispersed polymer standards with masses of 300–1500 *m*/*z*.

The conventional dried-droplet method was employed to spot 1 μL of 1 mg mL^–1^ sample solution on the stainless-steel sample plate; the solvent was then allowed to dry in air for several minutes. Using CS_2_ or toluene led to LDI mass spectra or similar quality. In a series of test measurements aimed at reducing supramolecular aggregation effects in the mass spectrometer,^50^ an excess of dithranol was added to the sample solutions to dilute the asphaltene and resin samples in the final precipitate. However, the recorded spectra did not show any significant effects from the addition of dithranol. While dithranol, a common MALDI matrix, may as well assist the ionization of the sample, this is uncertain in the present case, since a large fraction of the asphaltene and resin constituents efficiently absorb the laser light and similarly activate the desorption/ionization process.

### 2.6 Data Analysis

The peaks observed in the APCI-orbitrap mass spectra were assigned to molecular stoichiometries of the form C_*c*_H_*h*_N_*n*_O_*o*_S_*s*_, based on the recorded exact masses. Each peak in the spectrum was assigned to the stoichiometry that provided the best concordance with the experimental mass. The mass resolution and tolerance (5 ppm) of our mass spectrometer are at the limit required for the identification of sulfur, because of the small difference of ca. 0.003 Da between the masses of C_3_ and SH_4_. Consequently, sulfur was introduced in the fitting procedure only if no satisfactory mass match was otherwise obtained. The number of peaks assigned to compounds with one S atom was <2% in all cases and corresponded to compounds within the high-resolution end of the spectrometer (*m*/*z* < 400).

The molecular species thus identified were then classified in terms of their heteroatom content and aromaticity, as described by different parameters, including double-bond equivalents (DBEs) and Kendrick mass defects (KMDs). The DBE is defined according to the “nitrogen rule”:
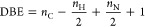
where *n*_C_, *n*_H_, and *n*_N_ denote the number of carbon, hydrogen, and nitrogen atoms in the molecule, respectively.^66,67^ The Kendrick mass scale assigns a full mass of 14 amu to ^12^CH_2_, and it is consequently related to the IUPAC ^12^C mass scale through a factor of 14.00000/14.01565.^68,69^ The Kendrick scale becomes particularly comprehensive when the analytes can be sorted out into families sharing heteroatom content and differing in the length of aliphatic chains, since the net incorporation or removal of CH_2_ groups does not alter the Kendrick mass defect. Conversely, the incorporation of ring units to condensed polyaromatic structures may be achieved with a change in mass equivalent to C_2_, C_3_H, or C_4_H_2_, involving changes in KMD of 0.027, 0.033, or 0.040 amu, respectively.

## 3 Results

### 3.1 Elemental Analysis

The results of the C, H, N, S elemental analysis of the asphaltene and resin fractions are summarized in Table 2. The *n*-hexane (A1) and *n*-heptane (C_7_-A1) asphaltenes from deposit DP1 show a similar percentage weight of C (∼88%), while A1 displays a roughly 20% greater heteroatom content (4.5% vs 3.75%). These values differ significantly from those observed for the *n*-hexane asphaltene from deposit DP2 (A2), which displays a remarkably low percentage weight of C (79.5%), which is indicative of a high heteroatom content. The elemental analysis yields similar values for sulfur in all of the asphaltenes, and a smaller abundance of nitrogen in asphaltene A2 vs asphaltene A1. Hence, a large oxygen content in asphaltene A2 (∼14%) emerges as the most plausible explanation for the small carbon weight. Unfortunately, we could not perform direct analysis of the oxygen content of our samples with the techniques at hand; the values in Table 2 are inferred from mass balance. While such a high oxygen content may seem unusual, similar values, ranging within 10%–14%, have been reported in previous analyses of downstream deposits of crude oil from different wells within the Hassi–Messaoud fields.^19,59^ The stage of processing or aging mechanism responsible for the oxygenation of the crude oil is under discussion with the engineers of the Sonatrach Company and will be the topic of future research.

The polar resin fraction, R2, consistently displays a greater heteroatom content than its less-polar counterpart resin R1 (∼12 wt % vs 3 wt %, respectively). Although the abundance of all heteroatoms increases in resin R2 vs resin R1, the main contribution is related to the oxygen content. Moreover, the net H/C values suggest that the A2 and R2 fraction exhibit higher aromaticity than their A1 and R1 counterparts.^70^

### 3.2 Aggregation Onsets

**Figure 2:**
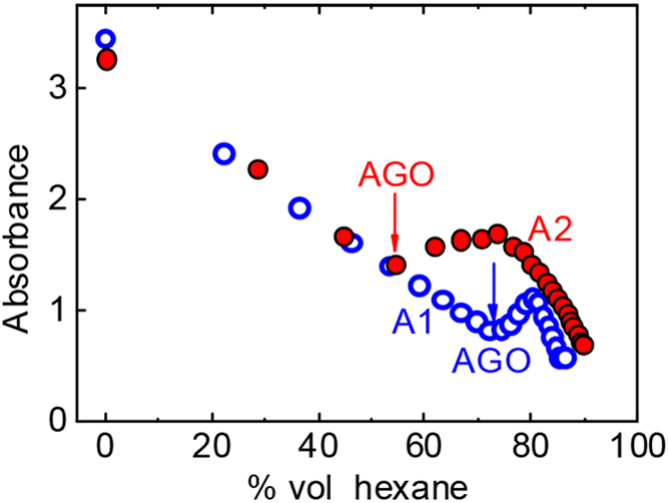
Determination of the aggregation onset (AGO) of asphaltenes A1 and A2 in 5 mg/mL toluene solution (open blue and solid red symbols, respectively). The absorbance at 750 nm is monitored as an increasing volume of hexane is added to the toluene solution, to induce precipitation.

The stability of the asphaltenes was assessed in terms of their precipitation propensity in toluene solution (5 mg/mL) with *n*-hexane as a flocculant. Figure 2 shows the change in absorbance at 750 nm, recorded as a function of added volume of *n*-hexane. The absorbance initially decreases because of dilution of the asphaltenes; the threshold for aggregation is detected as a sudden increase of the apparent absorbance associated with light dispersion, which defines the aggregation onset (AGO).^19^

The results indicate that the A2 asphaltenes are less stable (AGO = 55 vol % of *n*-hexane; precipitate content = 52 wt %) than the A1 asphaltenes (AGO = 72 vol % of *n*-hexane; precipitate content = 20 wt %). Note that the higher oxygen content of the A2 asphaltene correlates with a higher propensity to flocculate, in comparison to the A1 asphaltenes. This indicates that degradation/aging of the Hassi–Messaoud asphaltenes due to processing and storage has a particularly large effect on the formation of deposits.

### 3.3 APCI-Orbitrap Mass Spectra

APCI-orbitrap mass spectra were recorded for the asphaltene and resin samples in positive and negative ion mode, with the aim of monitoring the greatest diversity of molecular species possible and of highlighting the relative abundances of acidic (deprotonated negative ions) and basic (protonated positive ions) polar species. Recall that crude oil extracts are particularly complex materials, so information about their compositional landscape, provided by any instrumental technique, will be biased according to analytical responses. In the case of mass spectrometry, ionization efficiencies may vary over many orders of magnitude for different chemical families, depending on the type of source employed. For instance, electrospray ionization is most sensitive to polars and laser desorption ionization to polyaromatic species, with little efficiency for aliphatic compounds. The APCI source presently employed is most suitable for species of moderate size with medium to high polarity.^62^ Consequently, it has been shown that multiple separation stages of crude oil extracts may be required to have access to compounds with low relative analytical responses.^3,41^ This work constitutes a first incursion into the application of high-resolution mass spectrometry to the characterization of Algerian crude oils. Future studies will explore the implementation of specific sample pretreatment and differential precipitation procedures to further subfractionate the asphaltene and resin extracts.

**Figure 3:**
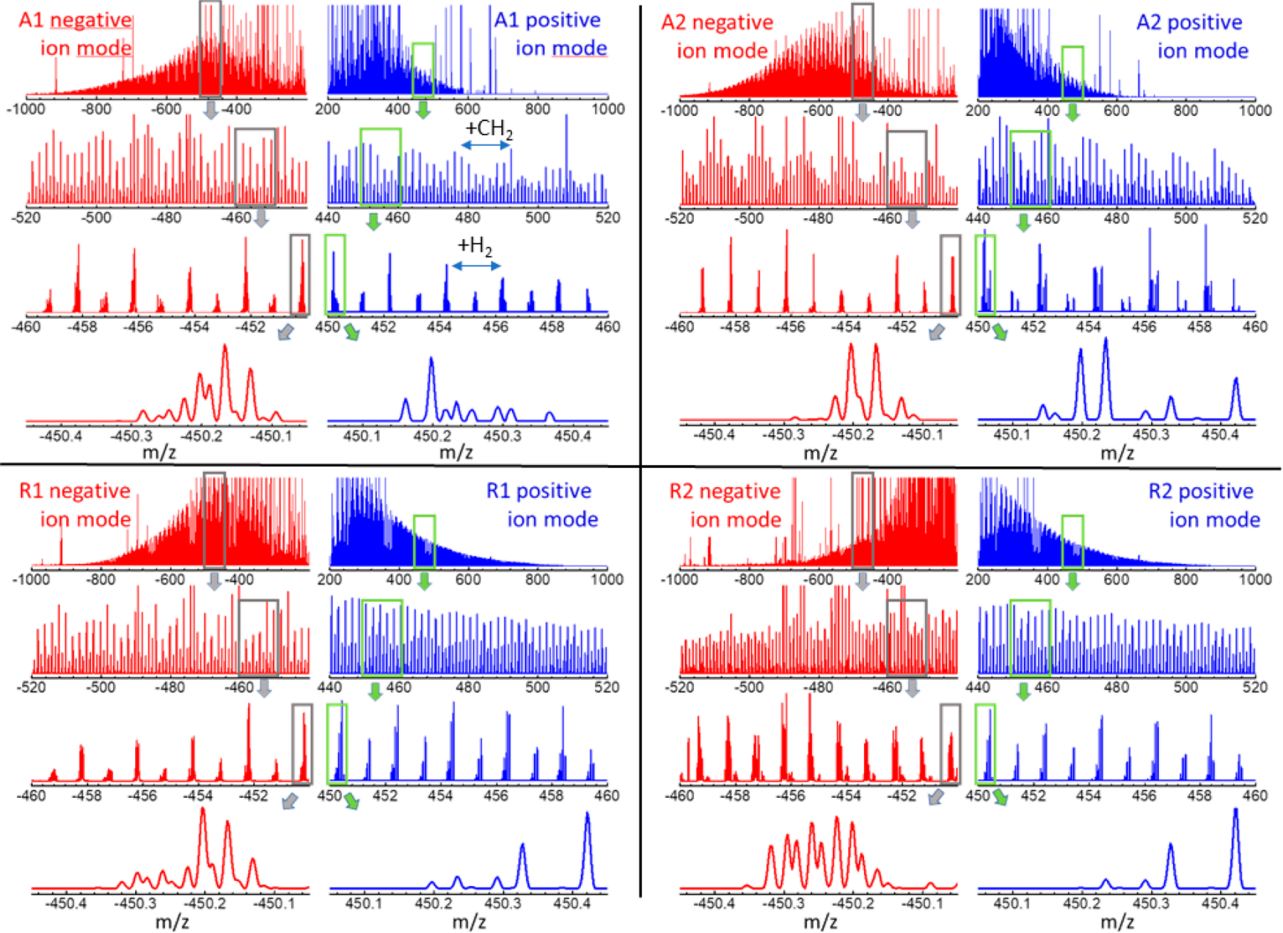
APCI-orbitrap spectra in negative- and positive-ion modes (red and blue traces, respectively), recorded for the asphaltene (A1, A2) and resin (R1, R2) extracts from Algerian crude oil. The complexity of the spectra is illustrated at different levels of amplification of the mass range. See Figures 4 and 5 for illustrative assignments of the observed peaks to molecular stoichiometries.

**Figure 4:**
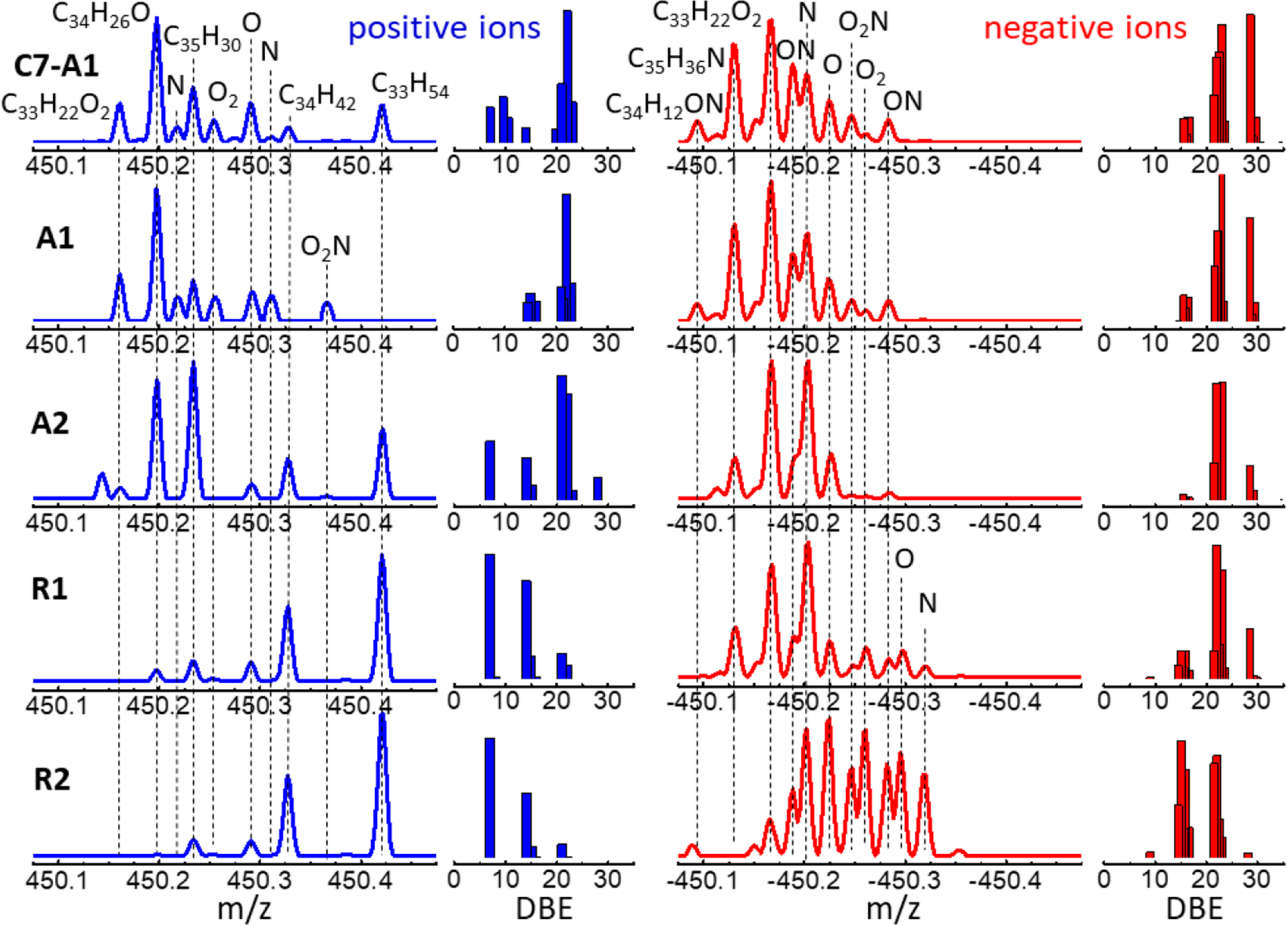
Illustrative assignment of chemical constituents in the APCI-orbitrap mass spectra of the asphaltene (C7-A1, A1, A2) and resin (R1, R2) samples. The heteroatom classes associated with the main peaks in the spectra are indicated; full stoichiometries are indicated for some of the peaks for reference. The bar diagrams represent the relative abundance versus the DBEs associated with the molecular species detected in each spectral window.

**Figure 5:**
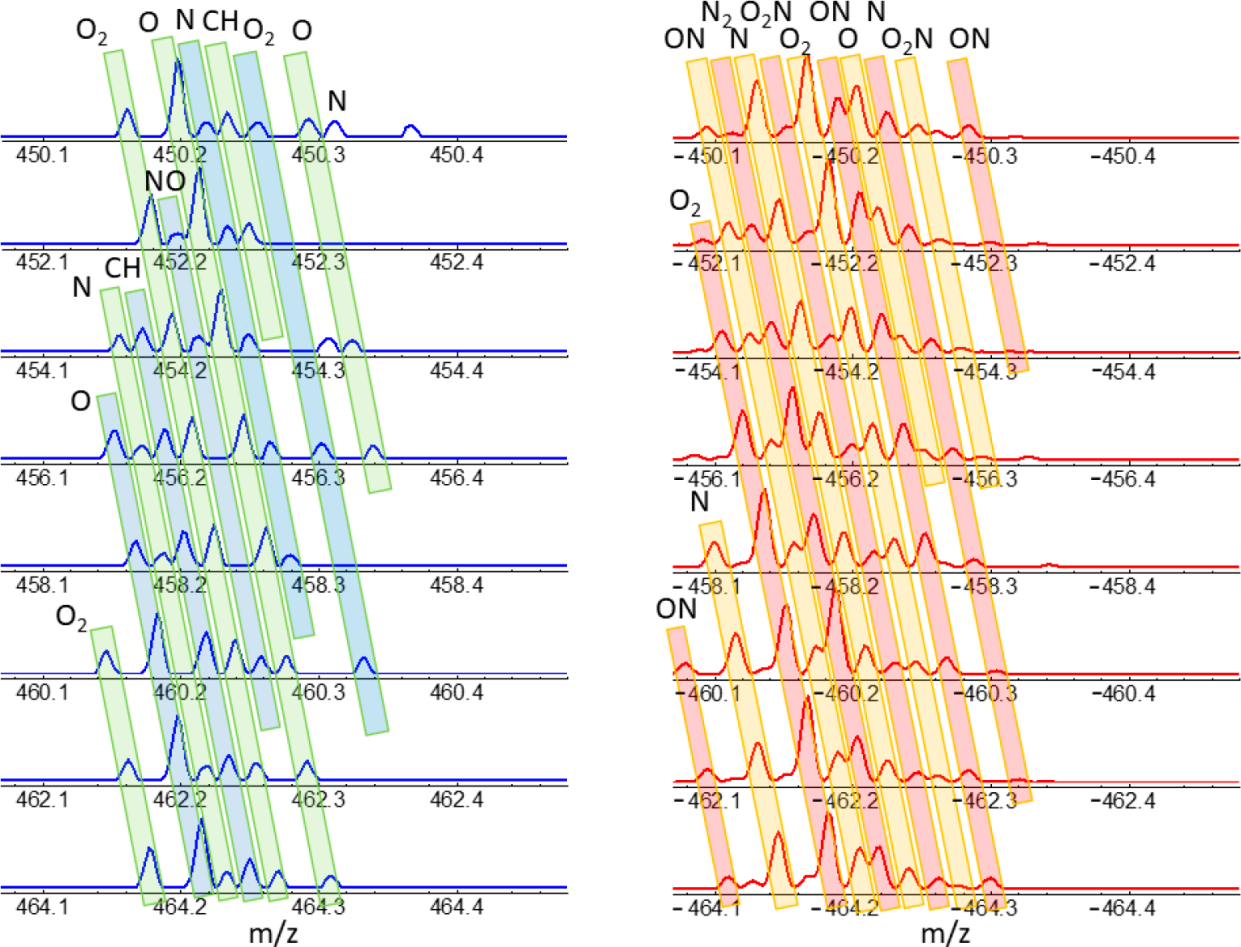
Identification of homologue heteroatom sequences illustrated for the APCI-orbitrap spectra recorded for the A1 asphaltene, in positive and negative ion modes (blue and red spectral traces, respectively). Spectra are shown in intervals of 2 mass units (loss or gain of two H atoms, associated with single-bond/double-bond substitutions in the molecular structure). The bands with alternating colors are meant to highlight the evolution of the peaks associated any given heteroatom class. Note the coincidence in the overall shape of the mass spectra separated by 14 nominal mass units (hence showing compounds with structures differing in one CH_2_ group; consequently, the spectra overlap if represented in terms of the Kendrick mass scale (not shown)).

The APCI-orbitrap mass spectra for the asphaltene A1 and A2, and resin R1 and R2 samples are displayed in Figure 3 at different degrees of magnification, to illustrate characteristic trends related to (i) the mass spread of the analytes over the 200–1000 *m*/*z* range; (ii) peak recurrences with 14 or 2 Da periodicities, related to the incorporation or removal of CH_2_ groups (length of side chains) or pairs of H atoms (number of double bonds), respectively; and (iii) the ensemble of peaks that are observed within any given nominal mass. Figures 4 and 5 provide examples of peak assignments, further illustrating the diversity of heteroatom classes observed in the crude oil extracts.

In general terms, the negative ions reach greater molecular weights than the positive ions. While the negative-ion spectra display analytes up to 1000 *m*/*z*, the positive-ion spectra have comparably weak signals above 800 *m*/*z*. This is plausibly related to the higher sensitivity of APCI for polar compounds. In addition, the negative-ion mass spectra has a tendency to detect a greater variety of molecular stoichiometries within each nominal mass (above 10 in most cases). Figure 4 lays out an illustrative assignment of the chemical classes (C_*x*_H_*y*_, O_*x*_, N_*y*_, N_*y*_O_*x*_) observed for each material in positive- and negative-ion modes. The positive-ion spectra incorporate a larger fraction of native hydrocarbons with C_*x*_H_*y*_ stoichiometries, while the negative-ion spectra capture compounds with higher heteroatom content. Measuring both ion polarities is key to exposing the different composition of the four samples through specific features and trends in the mass spectra. Note, for instance, that the two resin fractions produce similar mass spectra in positive-ion mode but display significantly different ensembles of chemical compounds in negative-ion mode. Conversely, the positive-ion spectra allow discerning asphaltenes from resins more neatly than, for instance, the negative-ion spectra of asphaltene A2 and resin R1. The higher degree of aromaticity of the asphaltenes over the resins is apparent from the DBEs of the molecular species observed within the 450–451 Da spectral window used for illustration. The overall DBE distributions are discussed below.

Figure 3 highlights collective peak position and intensity trends in the recorded mass spectra that are related to changes in length of the side chains and in the number of double bonds. Figure 5 illustrates this feature in greater detail by depicting the evolution of the mass spectrum of asphaltene A1, over 14 mass units. Each peak in the spectra is assigned to a given stoichiometry and the representation then indicates the steady increase of the mass defect with growing mass in each heteroatom class, as a consequence of the increased number of H atoms.

A noticeable similarity can be appreciated in Figure 5 between the spectra of analytes differing in the nominal mass of a CH_2_ group (e.g., *m*/*z* 450 vs 464, the mass spectra within these two nominal masses virtually overlap when represented in the Kendrick mass scale). All of these features are consistent with island- and archipielago-type molecular structures, combining polyaromatic cores with hydrocarbon chains.

**Figure 6:**
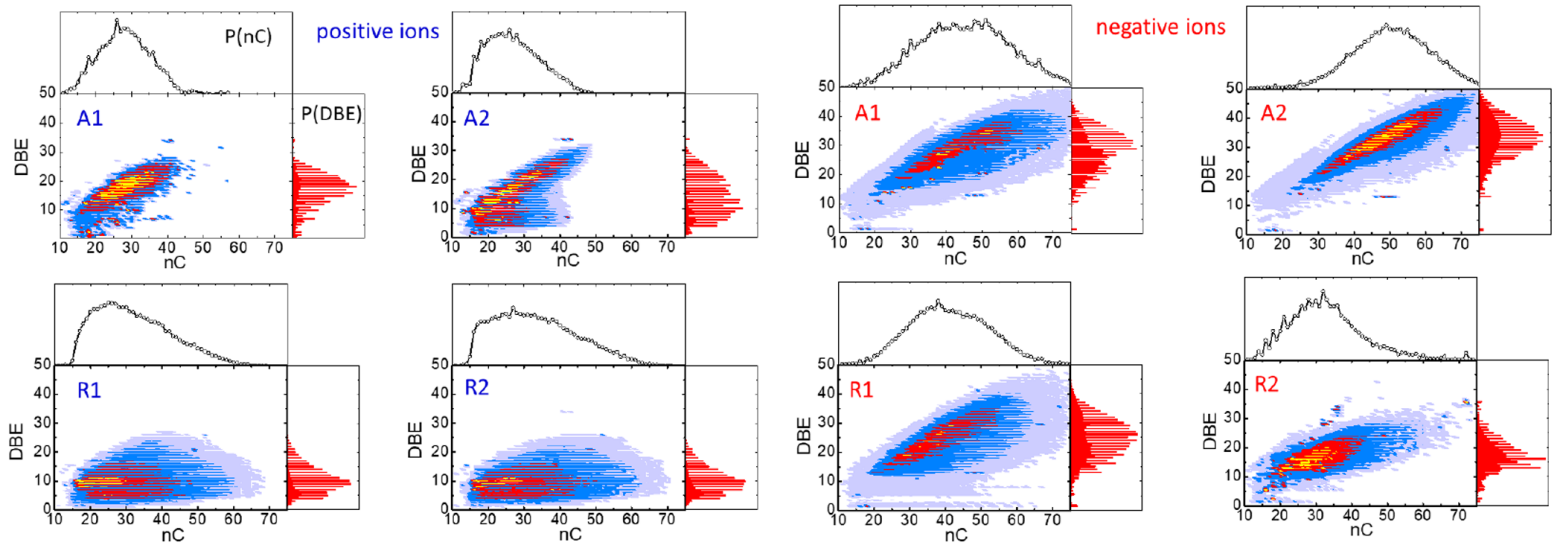
:Contour plots mapping the DBEs versus number of carbon atoms for the compounds assigned in the APCI-orbitrap spectra of the asphaltenes (A1, A2) and resins (R1, R2). The top and side layers of each frame depict the integrated carbon number and DBE probability distributions, respectively.

At this point, we consider the aromatic character of the four crude oil extracts in greater detail. The illustrative spectra and assignments outlined on Figures 4 and 5 show that asphaltenes are typically richer than resins in compounds with small mass defects and, correspondingly, higher DBEs. This already suggests higher abundances of polyaromatic moieties in their constituents. Figure 6 depicts contour plots of the DBEs versus the number of carbon atoms per molecule (*n*_C_), with color codes reflecting relative abundances. Such contour plots are commonly employed in modern petroleomics, because they provide compact overviews of large ensembles of chemical compounds, leading to convenient comparisons between samples.^2−5,66,67,71,72^

Figure 6 corroborates that DBE diagrams conform efficient signatures to discern asphaltenes from resins, as described in the following. The compounds detected in positive-ion mode display differentiated DBE trends in the asphaltene and resin samples. For the two resins, the DBE values observed are comparably low (<20), with an average value of ∼10. Moreover, this average value is weakly dependent on the number of carbons, which indicates that the growth in molecular size is associated with the incorporation of saturated aliphatic units to the molecular structure. In contrast, the DBE of the positive ions of the asphaltenes extends to 30 with a steady growth of the average DBE with the number of carbons (e.g., at *n*_C_ = 20 and 35, the average DBE values are 12 and 20 for asphaltene A1, and 10 and 24 for asphaltene A2, respectively). Hence, for the asphaltenes, the increase of carbon atoms in the molecules is largely associated with the growth of their polyaromatic cores. Aromaticity is consistently enhanced in the negative ions for the four samples, especially as the number of carbon atoms increases. This can be traced back to the fact that heteroatoms are primarily incorporated in aromatic moieties. Only the less-polar resin fraction R1 maintains a comparably low aromatic character, although even in this case, a neat positive increment of the DBE with *n*_C_ is appreciated. The A2 asphaltene fraction displays the most marked aromatic character, reaching DBE values close to 50 in the high *n*_C_ end of the distribution. This finding is consistent with the lower H/C ratio derived from elemental analysis (see Table 2).

**Figure 7:**
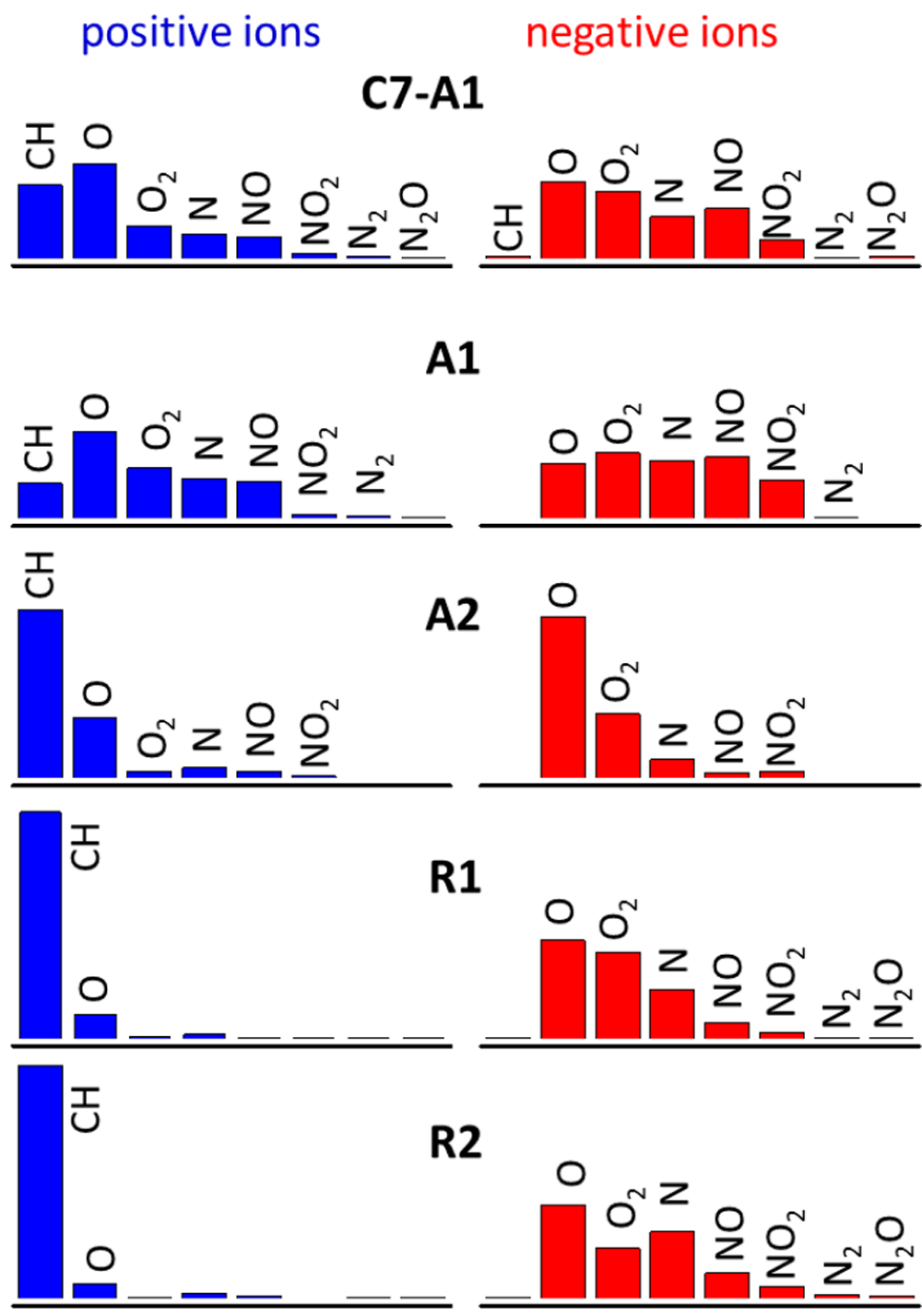
Relative abundances of the heteroatom classes, O_*x*_, N_*y*_, O_*x*_N_*y*_, and CH (no heteroatoms), as derived from the APCI-orbitrap mass spectra of the asphaltene (C7-A1, A1, A2) and resin (R1,R2) extracts.

To provide a compact characterization of the overall chemical composition of the crude oil fractions, the species identified in the APCI-orbitrap mass spectra are sorted out in Figure 7, according to their weighted average heteroatom content, into O_*x*_, N_*y*_, N_*y*_O_*x*_, and CH (no heteroatom) classes. This type of data reduction is commonly employed in petroleomics.^2−5^ In consonance with the illustrative mass spectral ranges discussed above, the chemical species detected in positive-ion mode display significantly lower heteroatom contents than their negative-ion counterparts. For the two resin fractions, roughly 90% of the compounds detected in positive-ion mode belong to the CH class, which contains no heteroatoms. Interestingly, the CH class is also dominant in the positive ions of the A2 asphaltene fraction (65%), although this asphaltene displays a significantly enhanced abundance of O_*x*_ species (23%), compared to the resin fractions (<10%). The positive ions of the A1 asphaltene are spread over different heteroatom classes; the O and O_2_ heteroatom classes are most abundant (ca. 47%), while the presence of CH compounds is appreciable as well (28%). The A1 products detected in negative-ion mode are richer in heteroatom content and further expose the chemical diversity of the components of the crude oil fractions. In this case, the dominant O and O_2_ species account for a joint abundance of 58% and most of the remaining 42% is distributed among the N and NO_*x*_ classes. In the A2 asphaltene in the negative-ion mode, the O and O_2_ classes are clearly dominant, with 87% of joint relative abundance. Hence, the A2 asphaltene is primarily conformed by polyaromatic hydrocarbons and their O-containing derivatives. Note that such marked abundance of O_*x*_ classes is consistent with the large O content, of up to 14 wt %, inferred from the elemental analysis of the A2 asphaltene (see Table 2). The R1 and R2 resin fractions both show broad distributions of heteroatomic classes, although with quantitative differences related to their different abundances in O_*x*_ compounds (70% in resin R1 vs 56% in resin R2) and in N-containing N/NO_*x*_ compounds (28% in resin R1 vs 42% in resin R2).

We close the discussion of the APCI-orbitrap spectra with considerations regarding the results for the *n*-heptane-extracted asphaltenes from deposit DP1 (C_7_-A1 asphaltenes), in comparison to the *n*-hexane asphaltenes A1 just discussed. Figure 4 illustrates the small differences observed in the mass spectra of the two asphaltene fractions. The negative-ion mode spectra are close matching over the whole spectral range, showing coincidence in the detected species with small quantitative variations in their relative intensities. The positive-ion spectra are as well largely coincident, although, in this case, some sizable differences in the ensemble of compounds detected in each fraction are observed, in particular for CH class compounds. The class distributions depicted in Figure 7 reveal that the abundance of heteroatomic species in both positive- and negative-ion modes is greater for the A1 asphaltene in comparison to the C_7_-A1 asphaltene, as would be expected for a *n*-hexane vs *n*-heptane extraction, although the difference is not large. These findings should be revisited in future investigations with a broader range of ionizing techniques (e.g., ESI and APPI).

### 3.4 LDI-TOF Mass Spectra

**Figure 8:**
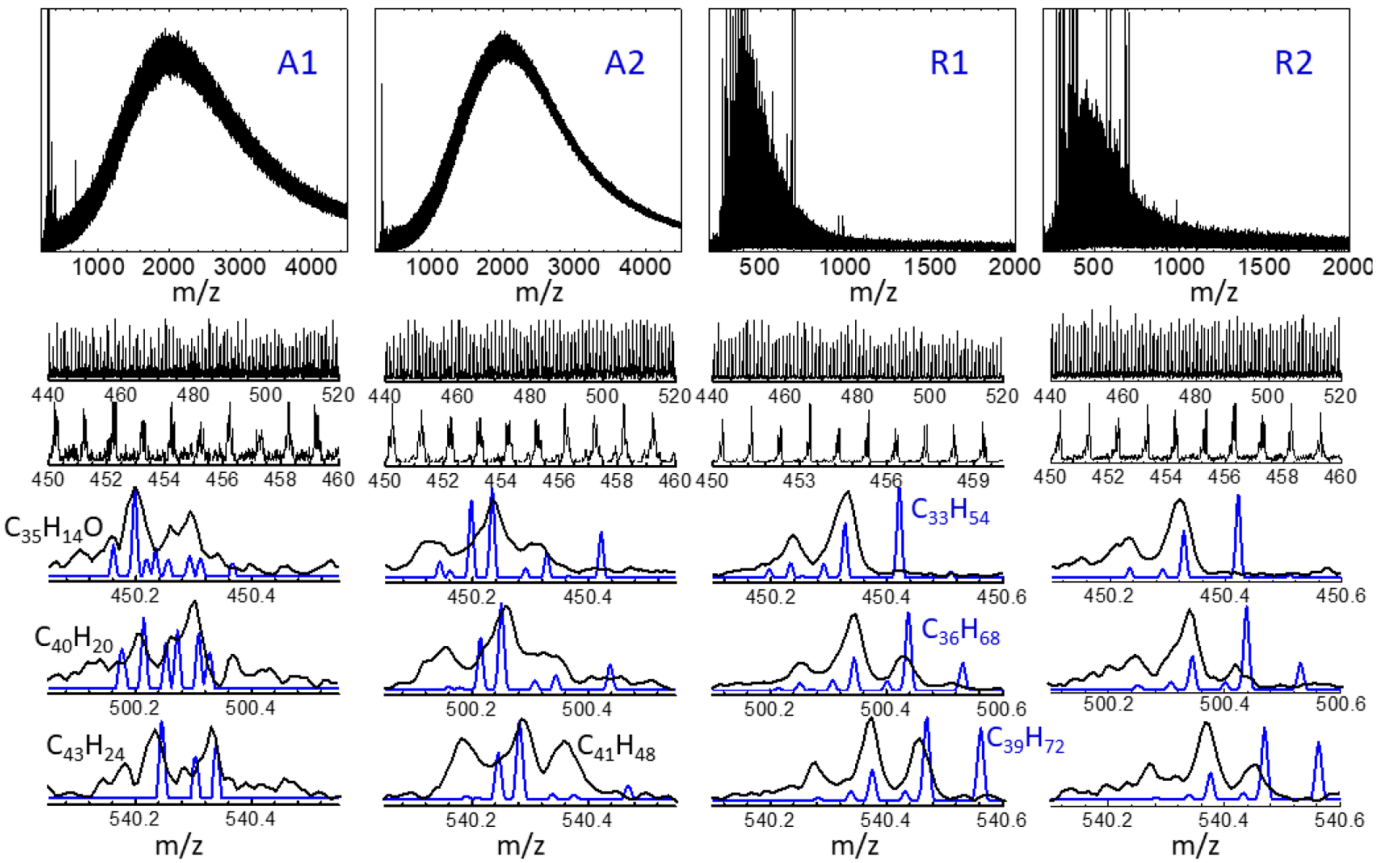
LDI-TOF spectra (black traces) of the asphaltene (A1, A2) and resin (R1, R2) extracts of crude oil. The spectra are shown at different levels of amplification of the mass range, in a similar way as in Figure 3. The three lower panels for each sample show a comparison with the corresponding APCI-orbitrap spectra (blue traces). The assignment indicated for some of the peaks serves to highlight the different ionization efficiencies of the LDI and APCI techniques (see text).

LDI-TOF mass spectrometry has been extensively used in petroleum analysis over the past two decades. As a main advantage, it achieves a soft ionization over a broad range of molecular weights for analytes embedded in light-absorbing matrices. LDI yields typically strong signals for crude oil extracts, because of their facile absorption of UV-vis or infrared (IR) light.^50,74−83^ A major challenge in LDI mass spectrometry analysis of asphaltenes is related to the difficulty of discerning between monomeric molecular species and their noncovalent aggregates. In LDI, the laser is typically applied to solid asphaltene or resin samples (in the form of powder, or of precipitate produced after evaporation of the solvent). Whereas the solid material sublimates upon laser heating, the density of the desorption plume is sufficiently high for many-body collisions to stabilize supramolecular aggregates as the plume expands and cools down. As a result, LDI mass spectra of crude oil extracts may extend over several thousand daltons. Figure 8 depicts the LDI-TOF mass spectra recorded for the asphaltenes and resins object of the present study. Asphaltenes A1 and A2 display broad LDI mass spectra, peaking slightly above 2000 Da and extending above 4000 Da. Resins R1 and R2 show narrower LDI distributions, although with long tails reaching 2000 Da. It is well-stablished that asphaltenes are more prone to aggregate than resins, which is consistent with the broader LDI mass distributions. Many works have reviewed comprehensively aggregation effects in LDI mass spectrometry of asphaltenes.^50,75,72−78^ This study will rather focus on the degree of coincidence in the molecular species detected with the LDI-TOF and the APCI-orbitrap techniques, in the mass region where both signals overlap.

Figure 8 presents a comparison of the LDI and APCI spectra measured for the four samples. An overall coincidence is found between the ion trends generated by the LDI and APCI techniques for the asphaltene and resin samples at molecular weights up to 600 Da. It can be observed that the LDI spectra display similar recurrent peak sequences as their APCI counterparts, with periodicities of 14 Da (CH_2_) and 2 Da (2H). Moreover, the detailed inspection over single nominal masses reveals that the position and relative intensities of the high-resolution orbitrap peaks are, to a large extent, consistent with the envelopes of the broader TOF peaks. Nevertheless, systematic differences are also appreciated between the spectra generated by the APCI and LDI techniques. On the one hand, LDI yields an excess of signal of products with small mass defects (<0.2 Da), hence, those associated with H-deficient polyaromatic core structures. This is, for instance, the case for the peaks tentatively assigned to C_35_H_14_O, C_40_H_20_, and C_44_H_22_ (with DBE = 29, 31, and 34, respectively) in the spectra of the two asphaltenes depicted in Figure 8. For the A2 fraction, the differences are more appreciable than for the A1 fraction, and they extend to products of lower aromaticity (e.g., C_41_H_48_, with DBE = 18). On the other hand, the mass spectra of the two resins suggest that LDI largely underestimates the peak intensities at high mass defects (>0.4 Da), which can be traced back to a low sensitivity toward compounds of low aromaticity and marked aliphatic character. In the illustrative examples of Figure 8, this is the case for the peaks assigned to C_33_H_54_, C_36_H_68_, or C_39_H_72_ (with DBE = 7, 3 and 4, respectively) in the mass spectra of the R1 and R2 resin fractions.

Summarizing, there is substantial overlap between the ensembles of molecular ions produced by the LDI and APCI techniques, up to molecular weights of ∼600 Da. The large optical absorbance of the resin and asphaltene matrices assists the ionization of a broad range of molecular components for which direct laser ionization is less efficient. Nevertheless, a trend for an enhanced ionization of polyaromatic (large DBE), and for a reduced sensitivity toward more aliphatic (small DBE) architectures, is observed in LDI vs APCI.

## 4 General Remarks and Conclusions

Asphaltene and resin fractions from Algerian Hassi–Messaoud crude oil have been characterized at a molecular level by means of high-resolution mass spectrometry. The asphaltene fractions are considered nonstable, with respect to precipitation, because they were extracted from two different deposits collected at the production and storage stages of the crude oil. The latter fraction (A2) is comparably less stable than the former fraction (A1), according to the lower aggregation onset observed in this study. The resin fractions were obtained directly from the crude oil and are considered as stabilizers regarding asphaltenes aggregation.

APCI-orbitrap mass spectrometry has been applied to characterize the molecular composition of the asphaltene and resin samples. The identification of positive and negative ionic species has served to expose a diversity of species with varying heteroatom content and overall basic and acidic character. In general terms, the present study extends earlier studies of the composition and structural properties of Algerian asphaltenes, using lower resolution mass spectrometry and spectroscopic methods (FT-IR and NMR),^57^ and corroborates the suggested changes in composition and aggregation propensity during the early stages of crude oil transportation and processing.

The main results of the present investigation may be summarized as follows:

• The deposit DP1 formed at the production well yields 88 wt % of *n*-hexane asphaltenes (A1) and is plausibly related to flocculation induced by the hydrocarbon content of the lift gas employed for enhanced oil recovery. According to the present analysis, the A1 asphaltenes display a broad heteroatom class composition, with a roughly even contribution from O- and N-containing compounds and an appreciable polyaromatic character (average DBE of 16 and 28, for the positive and negative ions, respectively).

• The asphaltene fraction from deposit DP2, formed at the downstream storage tank, yields 85 wt % of *n*-hexane asphaltenes (A2). A characteristic feature of the A2 asphaltenes is a large oxygen content, of as much as 14% according to the evidence provided by the elemental analysis. Consistently, the mass spectrometry analysis indicates a dominant abundance of the O-containing heteroatom classes (the O and O_2_ classes account for 80% of the negative ions detected). The processes leading to this apparent overoxygenation of the DP2 material are currently uncertain and will be a topic of future research. The A2 fraction also features a greater abundance of native hydrocarbons (CH class) and a significant overall polyaromatic character (average DBE of 15 and 33 for the positive and negative ions, respectively). The large O content and aromaticity correlate with a marked propensity for aggregation in the A2 asphaltenes, as derived from its comparably small aggregation onset, of 55 vol % of hexane/toluene solution versus 72 vol % for A1.

• The resin fractions of the Hassi–Messaoud crude oil are found to be abundant in aliphatic hydrocarbons and heteroatomic compounds of moderate aromaticity. The more polar resin fraction, R2, is enriched in O_*x*_ classes (56%) and markedly in N-containing species (42%), with respect to the less-polar resin fraction R1 (28%). These results suggest a stronger interaction of the R2 resin fraction with asphaltenes, potentially leading to enhanced stabilization effects, consistent with previous observations.^34^

The application of LDI-TOF mass spectrometry to the samples yields high mass signals that are plausibly related, to an uncertain extent, to supramolecular aggregates. Nevertheless, at low masses (<600 Da), the bands observed in the LDI-TOF mass spectra are, to a large extent, consistent with the ensembles of molecular ions produced with APCI. Nevertheless, the comparison of the spectra produced with two techniques suggests that LDI, in comparison to APCI, enhances the ionization of polyaromatic, plausibly “island-like”, compounds of asphaltenes with large DBE, whereas it yields a reduced sensitivity toward more aliphatic species, with saturated side chains and a comparably smaller DBE.

This study constitutes an approximation to the detailed composition of Algerian Hassi–Messaoud crude oil. Further investigation is required to progress in the elucidation of the molecular properties that affect its stability and the precipitation of its asphaltene fraction. In particular, detailed insights into the role of the dominant molecular structures in the aggregation of asphaltenes, e.g., island versus archipielago architectures, would require extensive subfractionation prior to mass spectrometry analysis and plausibly a combination of different ionization techniques.^3^ Future work in our group will follow that strategy and will furthermore investigate the selective precipitation and flocculation of asphaltene subfractions in the presence of resin fractions of different polarity.
